# Strategic contracting practices to improve procurement of health commodities

**DOI:** 10.9745/GHSP-D-14-00068

**Published:** 2014-06-24

**Authors:** Leslie Arney, Prashant Yadav, Roger Miller, Taylor Wilkerson

**Affiliations:** a University of Michigan, The William Davidson Institute, Ann Arbor, MI., USA; b University of Michigan, The William Davidson Institute, Ross School of Business, and School of Public Health, Ann Arbor, MI., USA; c LMI, McLean, VA., USA

## Abstract

Public-sector entities responsible for procurement of essential medicines and health commodities in developing countries often lack the technical capacity to efficiently ensure supply security. Under strict public scrutiny and pressures to be transparent, many agencies continue to use archaic procurement methods and to depend on inflexible forecasts and cumbersome tendering processes. On the basis of semi-structured literature reviews and interviews, we identified framework agreements as a strategic procurement practice used by the U.S. federal government that may also be suitable for global health supply chains. Framework agreements are long-term contracts that provide the terms and conditions under which smaller repeat purchasing orders may be issued for a defined period of time. Such agreements are common in U.S. and United Nations procurement systems and in other developed countries and multilateral organizations. In contrast, framework agreements appear to be seldom used in procurement of health commodities in countries of sub-Saharan Africa. The current practice of floating tenders multiple times a year contributes to long lead times and stock-outs, and it hampers the manufacturer's or supplier's ability to plan and respond to the government's needs. To date, government's use of strategic contracting practices in public procurement of health commodities has not received much attention in most developing countries. It may present an opportunity for substantial improvements in procurement efficiency and commodity availability. Enabling legislation and strengthened technical capacity to develop and manage long-term contracts could facilitate the use of framework contracts in sub-Saharan Africa, with improved supply security and cost savings likely to result.

## Abstract (precis)

Practices such as flexible, pre-established framework agreements can improve timeliness and cost of procurement and help improve commodity security. Addressing legislative barriers and building technical capacity in contract management may facilitate the use of such practices.

## BACKGROUND

Procurement and contracting play a significant role in determining the availability of, and thus access to, health commodities. The mean availability of many essential medicines in the public sector is lowest in the World Health Organization (WHO) Africa Region, followed by the WHO South East Asia Region, the regions which account for the majority of least-developed countries of the world.^1^^,^^2^ While different national procurement models exist across developing countries, procurement of essential medicines to serve many of these populations remains largely centralized in the Ministry of Health and/or a Central Medical Store (CMS) and relies heavily on public monies, international funding mechanisms, and donor funding.^3^ These public entities often lack the technical capacity to efficiently and strategically carry out the procurement process. Inadequate planning and forecasting, use of archaic procurement methods, and tendering yearly or multiple times a year contribute to high commodity costs, long lead times, stock imbalances, and, overall, commodity insecurity.^3^ Indeed, across all WHO regions, the mean availability of selected medicines is consistently lower in the public sector than in the private sector.^1^

An important outcome of the Paris Declaration on Aid Effectiveness was renewed focus on strengthening national procurement systems, as well as a commitment by donors to increase the use of country systems and procedures, such as national budgets and public financial management systems.^4^ In the last decade, many countries have seen the historical predominance of in-kind donations gradually replaced by direct budgetary support to governments.^3^ In other cases, donors have begun phasing out direct support to low-and middle-income countries (LMICs) who have graduated from low-income status.^3^ As a result of these shifts, many country governments have become increasingly responsible for the procurement of essential medicines and health care supplies.^3^

The private sector is often held up as a benchmark for efficiency for the public sector, but perhaps unfairly. Public-sector procuring entities face unique challenges and constraints, such as greater public scrutiny and lengthy procurement procedures. Corruption also presents a significant challenge, as some actors may encourage or maintain opacity to allow them to collect greater rents from the system. While transparency and corruption prevention are needed in the use of public monies, many feel that adopting additional checks and balances limits the agility and responsiveness of procurement practices. Even within the public sector, procurement of health commodities requires more flexibility and responsiveness to change (in population health and in environmental conditions) than procurement of other products.

Public-sector procuring entities face unique challenges and constraints.

The U.S. federal government, under public scrutiny in the use of public monies, is responsible for the provision of a large volume of health commodities. In 2012, the Department of Veterans' Affairs (VA) provided prescription drug coverage to 8.8 million military veterans, with prescription drug spending totaling approximately US$4.2 billion.^5^ In the same year, the Department of Defense (DOD) provided prescription drug coverage to 9.7 million active-duty and retired military personnel and their dependents, with spending totaling $7.6 billion.^5^ Provision of pharmaceuticals to a combined 18.5 million beneficiaries necessitates the use of strategic and efficient methods to control drug costs and ensure supply security. This study offers an overview of VA and DOD procurement and contracting practices and focuses on one strategic procurement and contracting practice that developing countries may benefit from adopting—framework agreements.

## METHODS

We conducted semi-structured literature reviews and interviews to identify strategic procurement and contracting practices of the U.S. DOD and VA that may be suitable for public procurement systems in developing countries. We reviewed key characteristics of these strategic practices as well as case studies of their use by other national governments and multilateral agencies. We then explored the public procurement legislation of selected countries of sub-Saharan Africa and evaluated the use and the challenges and barriers to use of these strategic practices in these settings. Much of the relevant literature on these topics is not published in peer-reviewed journals, but rather it is grey literature—presentations, websites, reports, government-issued assessments, and legislative documents. We obtained additional country-specific information through interviews and correspondence with persons involved in public procurement in selected countries of sub-Saharan Africa.

## FINDINGS

### U.S. Federal Government Procurement of Health Commodities

**Figure 1.**
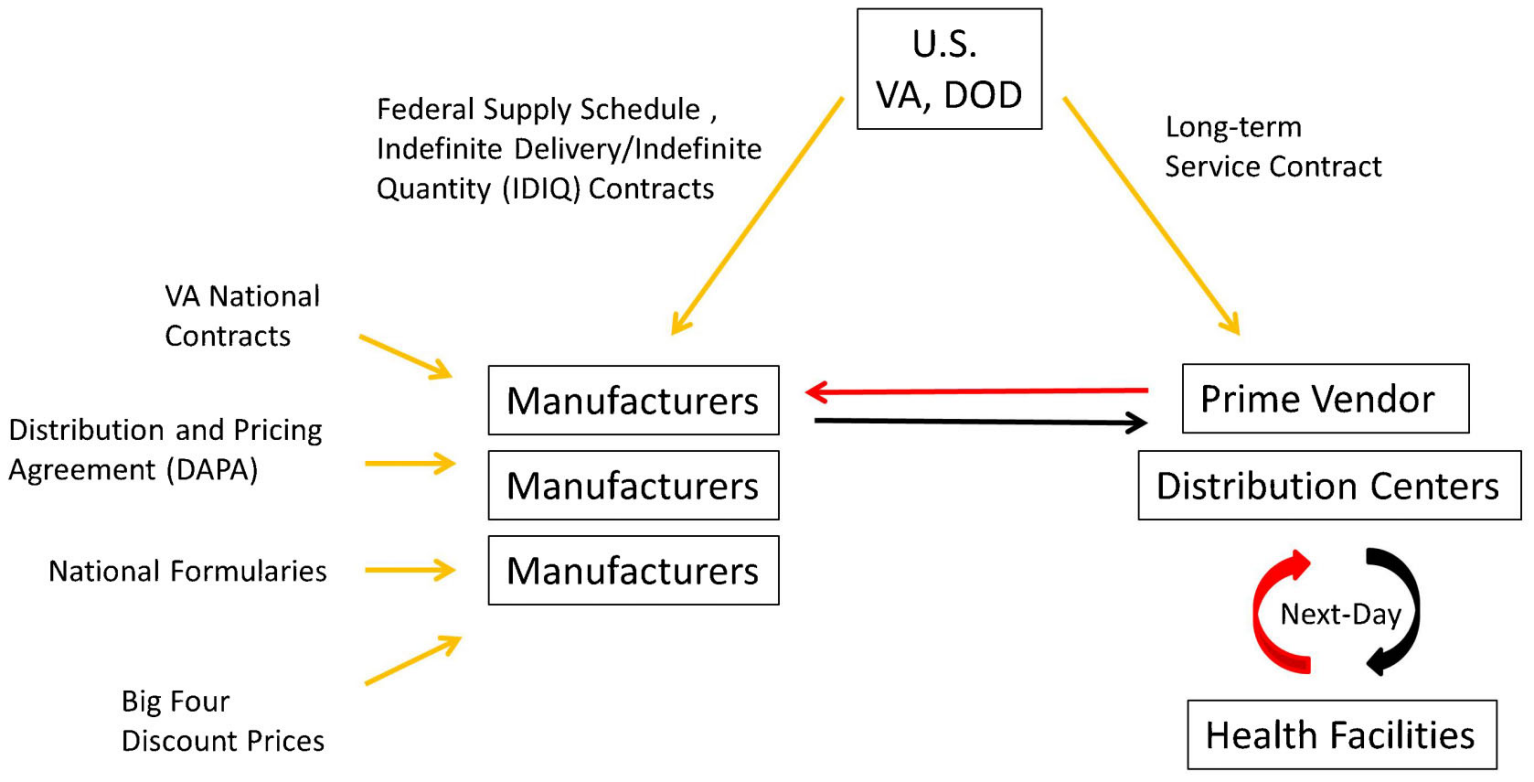
Overview of the U.S. Department of Veterans' Affairs and Department of Defense Procurement Systems for Essential Medicines Yellow arrows represent contracting or pricing arrangements, red arrows represent orders, and black arrows represent flow of supplies.

The VA and DOD procurement systems are generally characterized by centralized negotiation and contract management with decentralized purchasing/ordering authority. Key components of the 2 procurement systems include various federal pricing arrangements to control drug costs and the use of strategic contracting practices to maintain procurement flexibility. Direct purchase and distribution of commodities is then facilitated by prime vendor programs (Figure 1).

#### Pricing Arrangements

Under the rubric of centralized management and decentralized purchasing, a variety of federal pricing arrangements, among other mechanisms, help the DOD and VA control drug costs. First, the DOD and VA, along with the Public Health Service and U.S. Coast Guard (the so-called “Big Four” medical purchasers in the U.S. federal government), are eligible to receive federal ceiling prices, known as “Big Four” prices, on pharmaceuticals. These prices are statutorily mandated to be 24% lower than the manufacturer's average price for commercial customers.^6^ The DOD and VA also maintain prescription drug formularies, which help them obtain even more competitive prices from manufacturers for drugs included on the formularies.^6^

A variety of federal pricing arrangements help the DOD and VA control drug costs.

The DOD and VA receive discounts in return for commitment to vendors through a DOD- or VA-specific national contracts program.^5^ Similarly, the pricing of items procured by the Defense Logistics Agency (DLA) for DOD, as well as for other government military branches, takes place through direct negotiations with manufacturers through a Distribution and Pricing Agreement (DAPA).^7^ For DAPA pricing structures, vendors are allowed to unilaterally lower prices, usually to generate higher volumes and therefore improve market share.

#### Flexible Contracting Practices

The VA has the authority to establish the VA Federal Supply Schedule (FSS) for procurement of health care and medical commodities on behalf of all federal government agencies. Under the FSS, the VA negotiates firm, fixed-ceiling prices directly with manufacturers based on their most-favored commercial customer price.^8^ Through full and open competition, the VA establishes flexible multi-year contracts of indefinite delivery/indefinite quantity (IDIQ) with pre-approved suppliers under multiple-award schedules. VA Schedules are essentially catalogues of pharmaceutical products at prices available to all government agencies.^9^ Any agency's facilities can place orders directly with the prime vendors holding these Schedule contracts.

The VA and DOD gain procurement efficiency and added discounts through the Pharmaceutical Prime Vendor Program and the Medical/Surgical Prime Vendor Program, respectively. Prime vendors are preferred drug and medical supply distributors that facilitate the purchase of drugs and medical supplies by government facilities, followed by just-in-time (often next-day) delivery from a distribution center directly to the purchasing facility. Prime vendor programs shift inventory, inventory management, transportation, and personnel costs from the government to commercial firms.^7^ The VA and DOD also receive distribution fee discounts from their prime vendors. These are fixed percentage discounts off the lowest price available (FSS or Big Four).^7^

For prime vendor contracting, the United States is divided into regions, and regional contracts are awarded through a competitive process to the vendor, or combination of vendors, whose bid represents the best value for the government.^9^ It is important to note that prime vendors are not involved in the FSS or DAPA agreements or price negotiations established between the government and manufacturers. Although they may offer additional discounts, prime vendors are private wholesalers engaged in separate service contracts that facilitate the efficient ordering and delivery of the pharmaceutical products included under these government-wide framework contracts.

### Framework Agreements

As we have described, the use of flexible long-term framework agreements is a salient strategic practice of the VA and DOD procurement systems. We use the term **“framework agreement”** to describe **any contracting mechanism in which long-term contracts provide the terms and conditions under which smaller repeat purchasing orders (or call-off orders) may be issued for a defined period of time.**^10^ Different types of framework agreements may have different names depending on the context or legal system^11^—for example, long-term agreements (LTAs), task-order contracts, indefinite-quantity contracts, call-off contracts, umbrella contracts, rate or running contracts, system contracts, general service agreements, blanket purchase agreements, and standing offers.^10^^–^^15^

#### Two Stages

Framework agreements are typically comprised of 2 stages and can involve single or multiple suppliers. In a single-supplier framework agreement, a single contract is awarded to one supplier through a competitive process during the first stage of procurement, and then multiple call-off orders are placed directly against the contract throughout the duration of the agreement.^11^ In a multi-supplier framework agreement, a contract for the same good or service is signed with multiple suppliers in the first stage of procurement. The second stage of procurement in multi-supplier frameworks can be carried out in different ways: a secondary bidding process may take place for each call-off order, suppliers may have been ranked according to preference or capacity, orders may be rotated among the different suppliers, or fixed order amounts may be assigned to each supplier in the initial contract.^11^

#### Advantages

**Figure 2.**
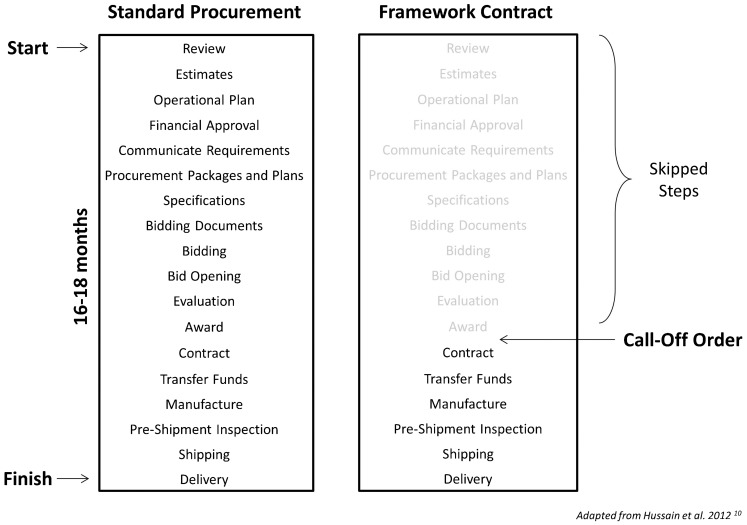
Framework Contracts Can Significantly Reduce the Number of Steps Involved in the Procurement Process

Framework agreements can save significant procurement time and resources by avoiding the repetition of all steps for each purchase (Figure 2).^10^^,^^11^ Entities can secure the benefits of centralized purchasing, through demand aggregation, while retaining flexibility in purchase quantities and delivery schedules. Framework agreements may also incentivize manufacturers or distributors to invest in assets (for example, equipment, personnel training, administrative, or operating procedures), which are specifically tailored to better serve government orders. The use of multiple-supplier framework agreements can help to ensure supply security, as a shortfall by one supplier can be compensated for or replaced by another supplier on the contract.^11^

With framework agreements, buyers secure the benefits of centralized purchasing while retaining flexibility in purchase quantities and delivery schedules.

### Case Studies of Framework Agreements

Various other national governments and multilateral organizations make use of framework agreements in diverse settings and procurement systems.

**Chile** created a government-wide e-procurement system, known as **ChileCompra**, in 2010 to enable government agencies to take advantage of the benefits of centralized purchasing without compromising the flexibility of decentralized ordering.^16^ Much like the VA and DOD procurement systems, ChileCompra negotiates multi-year agreements with suppliers for selected products. All government agencies can then order against these agreements using an electronic catalogue, receiving the lower prices negotiated by ChileCompra and avoiding the costs and lead times associated with floating individual tenders.^16^

In **Mexico,** the **State's Employees' Social Security and Social Services Institute** (Instituto de Seguridad y Servicios Sociales de los Trabajadores del Estado) (ISSSTE) is an important health service provider in Mexico's fragmented health-care system; it serves more than 12 million employees of the public sector and their families.^15^ In 2010, the Ministry of Public Administration (Secretaría de la Función Pública), which oversees public procurement in Mexico, initiated the use of framework agreements.^15^ As of 2012, ISSSTE had 10 framework agreements in place for the provision of various commodities including patented medicines, vaccines, vehicle maintenance, work wear, and personal protection equipment.^15^

The Joint Inspection Unit of the **United Nations** (UN) conducted an assessment in 2012 to evaluate the use, efficiency, and effectiveness of LTAs throughout the UN system. The assessment found that use of LTAs increased substantially between 2008 and 2011. The majority of UN organizations were realizing the benefits of LTAs, including the creation of administrative efficiencies and opportunities for greater volume leverage.^14^ The organizations making the most use of LTAs, as a percentage of total procurement, were the United Nations Children's Fund (UNICEF) at 93%, the United Nations Secretariat at 73%, and the United Nations Population Fund (UNFPA) at 45%.^14^ Both UNICEF and UNFPA *require* the use of global LTAs for the purchase of strategic goods, such as pharmaceuticals, vaccines and reproductive health products.^14^

Use of long-term agreements in the UN system increased substantially between 2008 and 2011.

The inspectors observed that there was no common definition or way to calculate the benefits or costs of LTAs within the UN system; indeed, with few exceptions, there was usually no calculation of the financial savings attributable to LTAs.^14^ Efficiency assumptions were often used in place of cost savings calculations.^14^

**UNICEF** establishes LTAs with manufacturers for the purchase of pharmaceuticals and vaccines following a competitive tendering process.^17^ The objective of these LTAs is to establish forecasts of quantities to be produced by the manufacturer(s) and quantities to be purchased in order to secure supply of the product over the duration of the agreement.^17^ UNICEF uses both single- and multiple-supplier LTAs to ensure greater supply security, and commonly splits arrangements to issue awards to multiple suppliers of each vaccine presentation.^14^^,^^17^ UNICEF procurement policies define 2 types of LTAs. Target-value LTAs are generally used for strategic essential supplies, are often split among multiple suppliers, and expire when either the maximum target amount or the date of contract expiry is reached.^14^ Time-bound LTAs are used when the unreliability of historical data and/or forecasted demand precludes creation of target-value LTAs; time-bound LTAs expire when the date of contract expiry is reached regardless of the volume procured.^14^ The duration for UNICEF LTAs has ranged from 1 to 10 years, with an average of 2 years plus a possible 1-year extension.^14^

Framework agreements are often seen—and feared—as anti-competitive. When a government engages in a framework contract with one manufacturer, other manufacturers may be discouraged from entering the market for the duration of the contract. UNICEF policies explicitly provide for the entrance of new manufacturers into the market in the middle of a multi-year tender.^17^ If a new manufacturer is not WHO-prequalified for the vaccine/product at the time of tender, the manufacturer must show a plan for obtaining prequalification.^17^ When the manufacturer obtains WHO prequalification, UNICEF considers awarding or reallocating a quantity to the manufacturer if UNICEF is facing a monopoly situation, if the current manufacturers' performance is inadequate, *or* if supply from the current manufacturer(s) is insufficient.^17^

**UNFPA**, as the largest procurer of reproductive health commodities, can achieve economies of scale and competitive prices on a variety of quality-assured products. UNFPA has established LTAs with more than 50 international manufacturers, with the intent to include all products under LTAs eventually.^18^ National governments, nongovernmental organizations, and other public-sector purchasers can take advantage of the competitive prices negotiated by UNFPA through the AccessRH portal, a UNFPA-managed procurement and information service for reproductive health commodities.^19^

The **Global Fund to Fight AIDS, Tuberculosis and Malaria** is another key global health stakeholder using framework agreements. A key component of the Global Fund's long-lasting insecticidal net (LLIN) strategy is a shift toward use of long-term contracts to optimize production, create a more sustainable market, and assist planning for manufacturing capacity. The bulk of the forecasted volume of LLINs for 2014 will be allocated using a 2-year LTA to multiple suppliers.^20^

The **Organisation for Economic Co-operation and Development** (OECD) conducted a survey on public procurement in 2012. Of the 32 OECD member countries responding to the survey, 31 reported routine use of framework agreements by some or all central-level procuring entities.^21^ About half of the OECD member countries were calculating the cost savings of the use of framework agreements, while a lack of data was commonly cited as the reason for not performing these calculations.^21^

### Framework Agreements in Selected Countries of sub-Saharan Africa

#### Legislative Provision

While there is no “single appropriate model” of public procurement, there has been a trend toward harmonization of public procurement procedures both within and across countries in an effort to promote international trade.^11^ First issued in 1994, the United Nations Commission on International Trade Law Model Law on Procurement of Goods, Construction and Services (UNCITRAL Model Law) was designed to help countries develop their public procurement systems and to provide a framework for procurement regulation.^11^ The Model Law serves as a template that national governments can flexibly use to reform or implement procurement legislation in accord with local circumstances and existing legislation.^22^ Generally, the Model Law promotes a procurement system based on a decentralized purchasing and decision-making mechanism but central regulatory or oversight authority.^23^ As of 2010, approximately 30 countries had enacted legislation based on the Model Law, including, in sub-Saharan Africa, The Gambia, Ghana, Kenya, Malawi, Nigeria, Rwanda, Tanzania, Uganda, and Zambia.^22^ Thus, the procurement laws of these countries share common guidelines and provisions but may differ on specific issues, such as value thresholds for permissible procurement methods.^23^ The list of countries may underestimate the influence of the UNCITRAL Model Law, as countries are not obligated to report adoption or use of the Model Law to the UN.^22^

There has been a trend toward harmonization of public procurement procedures to promote international trade.

The UNCITRAL Model Law of 1994 made no explicit mention of framework agreements, but the UNCITRAL Model Law of 2011 clearly outlines the conditions for use of framework agreements and corresponding procedures.^11^^,^^24^ According to the Model Law of 2011, a framework agreement procedure may be used when the procuring entity determines that the procurement need is expected to arise on a repeated, indefinite, or urgent basis during a given period of time.^24^

According to the UNCITRAL Model Law of 2011, a framework agreement procedure may be used when the procuring entity expects the need for procurement to arise on a repeated, indefinite, or urgent basis during a given period of time.

We reviewed the public procurement legislation and other official documents issued by the national procurement authorities of 7 sub-Saharan African countries for provisions concerning framework agreements (Table). The public procurement laws of Tanzania, Uganda, and Zambia explicitly provide for framework agreements.^13^^,^^25^^,^^26^ Although the laws of Ghana, Kenya, and Rwanda do not explicitly mention framework agreements, the procurement authorities of each country have issued other official documents or guidelines on the use of framework agreements.^27^^–^^29^ Mozambique's laws do not explicitly mention framework agreements, and we were unable to find supplemental documents or guidelines issued by the Government of Mozambique on their use.^30^

**Table:** TABLE. Summary of the Review of Public Procurement Legislation and Official Documents for Provision to Use Framework Agreements (FAs)

**Country**	**Procurement Authority**	**Legislation Governing Procurement**	**Legislative Provision for FA**	**Other Official Documents on FA**	**Terminology**
Ghana	Public Procurement Board	Public Procurement Act of 2003	No mention	Manual – Public Procurement Act of 2003	Framework (call-off) contract
Kenya	Public Procurement Oversight Authority	Public Procurement and Disposal Act of 2005	No mention	Public Procurement Manual for Health Sector 2009; The Public Procurement Guidelines for Framework Contracting 2010	Framework contract
Mozambique	Unit for the Supervision of Acquisitions	Decree No. 15/2010: Rules and Procedures on Procurement of Public Works, Supply of Goods and Services	No mention	—	—
Rwanda	Public Procurement Authority	Law n° 12/2007 of 27/03/2007 on Public Procurement	No mention	Intermediate Level Training Module in Public Procurement	Framework agreement (Indefinite Quantity Contract)
Tanzania	Public Procurement Regulatory Authority	Public Procurement Act of 2011	Yes	—	Framework agreement
Uganda	Public Procurement and Disposal of Assets Authority	Public Procurement and Disposal of Public Assets Act of 2003	Yes	The Public Procurement and Disposal of Public Assets Guidelines on Use of Framework Contracts 2011	Framework contract
Zambia	Public Procurement Authority	Public Procurement Act of 2008	Yes	—	Rate or running contract

#### Current Use

In **Zambia**, seeking to avoid the long lead times associated with international tenders, the Ministry of Health (MOH) in 2008 began creating flexible long-term contracts with national suppliers.^31^ Currently, the MOH is engaged in single-supplier framework contracts with 5 manufacturers or wholesalers for essential medicines from the Zambia National Essential Drug List, including antimalarial drugs, intravenous fluids, and various antibiotics for infectious diseases. These framework contracts are time-bound, with fixed volumes per product; they have a minimum duration of 2 years. Orders that have been forecasted are placed once a year, corresponding to budgetary allocation, and generally 4 call-off orders and deliveries take place per year per supplier. The use of framework agreements has added flexibility in quantities ordered and delivery schedules, increased the availability of medicines, and decreased stock-outs. The Zambia MOH has also seen an improvement in relationships with suppliers, additional transparency, and overall efficiency gains from the use of framework contracts (personal communication with Zambia Drug Supply Budget Line, September 2013).

The Zambia experience shows that a range of political, legal, and economic factors must be in place before framework contracts can be successfully used. The sequencing of activities in procurement reform is important to ensure that framework contracts are not used prematurely, which could contribute to the opacity of procurement practices. It is important, before framework contracts are used, first to establish a platform for monitoring procurement and contracts. This platform can share procurement information with responsible civil society groups and help enhance transparency and accountability in the procurement system, leading to greater trust and confidence in the procurement system.

In 2008, the **Ghana** Health Service was in the process of establishing National Framework Agreements with local private-sector suppliers in order to use the central-level contracting capacity to negotiate lower prices for the decentralized procuring entities.^31^ Time-bound framework agreements are currently in place for procurement of antiretroviral medicines. Although the benefits in terms of commodity assurance far outweigh the potential disadvantages, there have been problems with suppliers' adherence to shipment schedules, which have led to overstocking, expiries, or shortages (personal communication with Ghana Ministry of Health, October 2013).

The **Kenya** Medical Supply Agency (KEMSA) is a parastatal organization mandated to manage the forecasting, procurement, warehousing, and distribution of essential medicines and health commodities to the population of Kenya. In the country's newly devolved health system, the Government of Kenya will begin allocating health budgets to county governments, which will purchase essential medicines and health commodities from KEMSA or (possibly) other sources. Aided by the creation of a contract management department within the organization, KEMSA has recently begun using framework agreements for procurement of all health commodities funded by the government. These 2-year framework contracts with domestic suppliers are of indefinite quantity at fixed prices. Each quarter, KEMSA issues forecasts and orders for the estimated quantities needed; payment is made on delivery (personal communication with KEMSA, March 2014).

The Secretariat Procurement Unit of the **Southern African Development Community (SADC)** manages a database of approved suppliers and places purchase orders under multiple framework contracts.^32^ SADC engages in pooled procurement, whereby Member States purchase directly from prequalified regional suppliers holding framework contracts.^33^

#### Barriers to Use

**Lack of explicit legislation.** In **Mozambique,** the public procurement legislation, Decree 15, does not explicitly mention framework agreements, and we learned from correspondence with the Central de Medicamentos e Artigos Médicos (CMAM), the CMS of Mozambique, that Decree No. 15/2010 does not allow for use of framework agreements. The lack of explicit legislative provision for framework agreements may constitute a barrier in other countries as well.

**Lack of technical capacity.** Engaging successfully in framework agreements requires adequate financial and human resources, including technical capacity in contract management and the ability to continually prepare, negotiate, manage, evaluate, and conduct performance reviews. A general lack of technical capacity at both the national and sub-national procuring entity levels has often been cited as a barrier to more efficient procurement practices and supply security.^3^^,^^34^^,^^35^ In Zambia, establishing a platform for procurement and contracts monitoring was a necessary first step in the adoption of framework agreements. Similarly, the creation of a new contracts management department within KEMSA was cited as essential to the adoption and implementation of framework agreements (personal communication with KEMSA, March 2014).

**Other issues**. Additional concerns about framework agreements, which may act as barriers to their introduction and use, include price volatility, local manufacturers' participation, and the inclusion of new technology during the course of the framework contract. Concerns regarding the timeliness of payment from procurers (that is, payment discipline) can also deter manufacturers from engaging in framework agreements.

As mentioned, multi-supplier framework agreements involve 2 stages and varying levels of competition. To deal with volatile markets, framework agreements may exclude prices from the terms and conditions agreed upon in the first stage of competition. Call-off orders may then be allocated to suppliers through a mini-competition at revised, current prices.^11^ Procuring entities in the UN system mitigate the risks of price volatility to LTAs by expressing the price as a fixed percentage discount off the supplier's catalogue price.^14^

As for local manufacturers, because call-off orders are of smaller volumes than bulk procurements and are spread over a longer period, multi-supplier framework agreements may promote participation of local manufacturers or of small and medium enterprises (SMEs) by rotating call-off orders among the suppliers.^11^ Target-value (volume-based) LTAs also may be split among multiple suppliers, with an appropriate, capacity-based volume allocated to local suppliers.

As in the U.S. government's prime vendor programs, the success of a framework contract depends on the availability, accuracy, and timeliness of shared data to improve the synchronization of public, donor, and supply chain systems. Additionally, while framework contracts can enable decentralized execution (ordering), they do not guarantee it; some framework contracts are used to support CMSs or other public supply chain organizations that do not delegate ordering functions to the local level.

Flexibility and responsiveness in the procurement of health commodities are especially important to take advantage of new technology. To promote open competition and the overarching goal of improving health, framework agreements for health commodities must consider provisions for the entry of new suppliers into the market during the course of an existing framework contract. UNICEF procurement policies allow for the entrance of new manufacturers in the middle of a multi-year tender, but the set of conditions permitting this does not specifically include emergence of new technology, products, or competitors. Framework agreements do effectively lower the levels of competition *within* the contracted period. Therefore, they may not be suitable in markets where new suppliers are likely to enter within the duration of the agreement. In this regard, the use of framework agreements is better suited for products with more mature markets.

## DISCUSSION

With adequate technical capacity, shared information, and appropriate legal provisions, framework agreements can allow for flexibility and responsiveness in ordering and delivery while maintaining transparency and achieving greater value-for-money in the procurement of essential medicines and health commodities. In assessing the public procurement systems of 2 U.S. federal agencies involved in buying health products, we identified the use of centralized framework agreements as a key factor in retaining flexibility in procurement while controlling drug costs. Framework agreements also are widely used in the UN system and by most OECD member nations, perhaps reflecting the level of technical capacity in procurement more commonly found in global agencies and developed countries.

Framework agreements can allow for flexibility and responsiveness in ordering and delivery while maintaining transparency and achieving greater value-for-money.

### Limited Use in Africa

In contrast, the use of framework agreements in the public procurement of health commodities in sub-Saharan Africa appears to be limited. While we did not explore the procurement legislation and systems of all countries of sub-Saharan Africa, the countries selected provide insight into the general use and barriers to use of framework agreements in the region. To varying degrees, Ghana, Kenya, and Zambia have adopted framework agreements for procurement of selected essential medicines. In some instances, however, the lack of enabling legislative may be a significant barrier to use, as in Mozambique. Still, the procurement laws of both Ghana and Kenya also do not explicitly mention framework agreements, but supplemental guidelines or manuals on their use have been issued by each country's procurement authority. Inadequate understanding or differing interpretation of public procurement legislation may impede the use of framework agreements and other strategic procurement and contracting practices, especially for countries that have undergone recent legislative reform. Insufficient technical and contract management capacity is commonly cited as a weakness of national procurement systems and may constitute a salient barrier to the use of strategic contracting practices by many developing countries.

Similarly, in many countries of sub-Saharan Africa, there are currently few well-developed, high-quality distributors that can be engaged as prime vendors to facilitate the direct purchase and distribution of commodities ordered from framework contracts. As the health commodity distribution market develops, it will be important for governments to explore the use of prime vendor arrangements for distribution.

The use of framework agreements does not, in and of itself, guarantee their benefits. Discretion in the use of framework agreements, strategic planning in the formulation of the agreement, sufficient contract management, and continual evaluation all are required to use a framework agreement in a way that preserves flexibility, achieves greatest value for money, and ensures supply security. Also, the use of framework agreements is not without risks. Given the smaller size of call-off orders, it may prove challenging to monitor the awarding of all call-off orders for legal violations, creating risks to competition and transparency.^11^ Therefore, corruption mitigation actions, such as counter verification mechanisms, must accompany the use of framework agreements.

The impact of framework agreements on SME participation and performance will also depend upon how the agreement is designed and operated.^11^ While some framework agreements can promote SME participation, the aggregation of smaller purchases can put SMEs at a competitive disadvantage.^11^ Furthermore, framework agreements can make it more difficult for SMEs to estimate costs, given that many work on a fairly small purchasing cycle due to lower credit availability. If limited credit or working capital constrains an SME from importing or producing the required quantities in a timely manner, missed deliveries—and, thus, stock-outs—could result. Good enforcement of service level agreements, and penalties and flexibilities embedded in the long-term agreement, are critical to ensure that there are no negative impacts for procurers. Mechanisms to increase credit availability to SMEs can help improve timeliness of deliveries and supply security and mitigate negative impacts for SMEs.

### Recommendations

Additional and more comprehensive research on the use of framework agreements for the public procurement of health commodities in developing countries is warranted. Highlighting successful use of framework contracts in sub-Saharan Africa may encourage additional countries to adopt more strategic contracting practices. A first step for all countries not currently using framework agreements should be to thoroughly examine national public procurement legislation. For countries without legislative provision for framework agreements, we recommend that public procurement authorities work toward legislative reform that includes such provisions in public procurement legislation. Where enabling legislation is in place, we encourage procuring entities to work to strengthen technical and contract management capacity and to consult stakeholders with experience and expertise in the use of framework agreements. A robust procurement organization in which framework contracts can be used requires 2 strong parts—procurement people and procurement procedures. Developing procurement human capital in ministries of health will help to promote greater use of framework contracts and will have broader benefits from more effective and efficient procurement in general. Organizations such as the Chartered Institute of Purchasing and Supply (CIPS) and People that Deliver can act as resource partners for such capacity-building efforts.

**Technical working group.** An international technical working group would be well-positioned to help developing countries adopt and manage framework agreements for procurement of health commodities. The technical working group could be constituted under the Interagency Pharmaceutical Coordination group (IPC) and composed of international agencies (for example, WHO, the United Nations Development Programme, UNICEF, the World Bank, the African Development Bank, and the Global Fund), developing-country ministries of health, and individuals with expertise and experience in framework contracting for pharmaceuticals and other health products. The aim of the technical working group could be to support procurement departments in ministries of health or medicines supply agencies in developing countries in the use of framework contracts. More specifically, the technical working group could:

- Provide technical leadership in developing framework contracts as a procurement approach
- Develop new knowledge resources to fill information gaps related to the use of framework contracts
- Develop and implement a strategic plan for promoting the use of framework contracts wherever suitable
- Design a workshop for developing-country procurers to disseminate information about the value of framework contracts.

## Notes

**Peer Reviewed**

**First published online:** 2014 Jul 24

## References

[1] Cameron A Ewen M Ross-Degnan D Ball D Laing R Medicine prices, availability, and affordability in 36 developing and middle-income countries: a secondary analysis. Lancet. 2009;373(9659):240–249 10.1016/S0140-6736(08)61762-6 19042012

[2] The World Bank: working for a world free of poverty [Internet]. Washington (DC): The World Bank Group; c2014 [cited 2014 Jun 22] Least developed countries: UN classification; [about 4 screens]. Available from: http://data.worldbank.org/region/LDC

[3] World Health Organization (WHO). The world medicines situation 2011. Geneva: WHO; 2011 Available from: http://www.who.int/medicines/areas/policy/world_medicines_situation/en/

[4] OECD Task Force on Public Financial Management. What are the benefits of using country systems? Paris: OECD; 2008 Available from: http://www.oecd.org/dac/effectiveness/48780908.pdf

[5] United States Government Accountability Office (GAO). Prescription drugs: comparison of DOD and VA direct purchase prices. Washington (DC): GAO; 2013 Available from: http://www.gao.gov/assets/660/654019.pdf

[6] United States Government Accountability Office (GAO). Testimony before the Subcommittee on Federal Workforce, Postal Service, and the District of Columbia, Committee on Oversight and Government Reform, House of Representatives. Prescription drugs: overview of approaches to control prescription drug spending in federal programs. Washington (DC): GAO; 2009 Available from: http://www.gao.gov/new.items/d09819t.pdf

[7] Directorate of Medical Materiel Defense Logistics Agency Troop Support. DAPAs made easy: a guide to DAPAs and the Medical/Surgical Prime Vendor Program. Directorate of Medical Materiel ; 2011 Available from: https://www.dmsb.mil/refDocs/final%20DAPAs%20Made%20Easy.pdf

[8] U.S. Department of Veteran Affairs [Internet]. Washington (DC): U.S. Department of Veteran Affairs; [last updated 2014 Jun 11; cited 2014 Jun 22] VA federal supply schedule service [about 2 screens]. Available from: http://www.fss.va.gov/

[9] Congressional Budget Office (CBO). The Department of Veterans Affairs' Pharmaceutical Prime Vendor Program. Washington (DC): CBO; 2009 Available from: http://www.cbo.gov/sites/default/files/cbofiles/ftpdocs/100xx/doc10009/02-25-va_vendor_letter.pdf

[10] Hussain Z Tukai M Adu JA Khan AI Workshop on framework agreement and two-year procurement cycle at Proshika HRDC, Koitta, Manikgonj, March 6–8, 2012. Arlington (VA): Management Sciences for Health; 2012 Available from: http://siapsprogram.org/wp-content/uploads/2012/09/12-117-Report-Framework-Workshop-format.final_.pdf

[11] Arrowsmith S Treumer S Fejø J Jiang L Public procurement regulation: an introduction. EU Asia Inter-University Network for Teaching and Research in Public Procurement Regulation ; 2011 Available from: http://eprints.nottingham.ac.uk/1689/1/eprintspublicprocurementregulationintroduction.pdf

[12] UNICEF. Procurement process for pharmaceuticals. Consultation with pharmaceutical manufacturers: 27th–28th September 2010. New York: UNICEF; 2010 Available from: http://www.unicef.org/supply/files/UNICEF_contracting_process__Francisco_Blanco.pdf

[13] Government of Zambia. The Public Procurement Act, 2008, No. 12 of 2008 309. Available from: http://www.zppa.org.zm/download.php?file = public_procurement_act_2008.pdf

[14] Terzi C Callejas JF Review of long-term agreements in procurement in the United Nations system. Geneva: United Nations Joint Inspection Unit; 2013 Available from: https://www.unjiu.org/en/reports-notes/JIU%20Products/JIU_REP_2013_1_English.pdf

[15] Organisation for Economic Co-operation and Development (OECD), Public Governance Committee. OECD public procurement review of the Mexican Institute of Social Security and Services for State Workers (ISSSTE). Paris: OECD; 2013 Available from: http://www.oecd.org/officialdocuments/publicdisplaydocumentpdf/?cote = GOV/PGC/ETH(2013)4&docLanguage = En

[16] Bornbusch A Bates J Multiplicity in public health supply systems: a learning agenda. Glob Health Sci Pract. 2013;1(2):154–159 10.9745/GHSP-D-12-00042 PMC4168568 25276528

[17] UNICEF Supply Division. Overview of UNICEF procurement processes: industry consultation. Copenhagen (Denmark): UNICEF Supply Division; 2012 Available from: http://www.unicef.org/supply/files/1._Overview_of_UNICEF_Procurement_Processes.pdf

[18] UNFPA [Internet]. New York: UNFPA; c2008 [cited 2014 Jun 23] UNFPA procurement—supplies for reproductive health results; [about 3 screens]. Available from: http://www.unfpa.org/public/procurement

[19] AccessRH [Internet]. Copenhagen (Denmark): UNFPA; c2008 [cited 2014 Jun 23] AccessRH: access to reproductive health supplies starts here; [about 2 screens]. Available from: http://www.myaccessrh.org/about-us

[20] Global Fund to Fight AIDS, Tuberculosis and Malaria. The Global Fund's LLIN procurement strategy, tender process and future plans: Global Fund/UNICEF pre-tender briefing, Copenhagen, 20th August 2013. Geneva: Global Fund; 2013

[21] Organisation for Economic Co-operation and Development (OECD), Public Governance Committee. Government at a glance 2013: procurement data. Paris: OECD; 2013 Available from: http://www.oecd.org/officialdocuments/publicdisplaydocumentpdf/?cote = GOV/PGC/ETH(2013)2&docLanguage = En

[22] Nicholas C Work of UNCITRAL on government procurement: purpose, objectives, and complementarity with the work of the WTO. 2011 Available from: http://bit.ly/J9DQr5

[23] World Health Organization (WHO); Management Sciences for Health; John Snow, Inc. A situational analysis and feasibility study on regional pooled bulk procurement of essential medicines and other health supplies in the East African community partner states: final report. Geneva: WHO; 2007 Available from: http://apps.who.int/medicinedocs/documents/s18414en/s18414en.pdf

[24] United Nations Commission on International Trade Law (UNCITRAL). UNCITRAL model law on public procurement. New York: United Nations; 2014 Available from: http://www.uncitral.org/pdf/english/texts/procurem/ml-procurement-2011/2011-Model-Law-on-Public-Procurement-e.pdf

[25] The United Republic of Tanzania. The Public Procurement Act, 2011, No. 9. Available from: http://www.gpsa.go.tz/images/pdf/act.pdf

[26] Government of Uganda. The Public Procurement and Disposal of Public Assets Act, 2003. Available from: http://opm.go.ug/assets/media/resources/365/The_Public_Procurement_And_Disposal_Of_Public_Assets_Act,_2003.pdf

[27] Rwanda Public Procurement Authority (RPPA). Intermediate level training module in public procurement. Kigali: RPPA; 2012 Available from: http://www.rppa.gov.rw/fileadmin/files/CapacityDev/Draft%20Intermediate%20Module.pdf

[28] Public Procurement Oversight Authority (PPOA) [Kenya]. Public procurement manual for health sector. Nairobi: PPOA; 2009 Available from: http://www.ppoa.go.ke/downloads/Manuals/procurement_manual_health.pdf

[29] Ghana Public Procurement Board. Manuals—Ghana Public Procurement Act, 2003 (Act 663).

[30] Government of the Republic of Mozambique. Decree No. 15/2010.

[31] Dalberg Global Development Advisors; MIT-Zaragoza International Logistics Program. Private sector role in health supply chains: review of the role and potential for private sector engagement in developing country health supply chains. New York: Rockefeller Foundation; 2008 Available from: http://healthmarketinnovations.org/sites/default/files/Private%20Sector%20Role%20in%20Supply%20Chains.pdf

[32] Southern African Development Community (SADC): towards a common future [Internet]. Gaborne (Botswana): SADC; c2012 [cited 2014 Jun 23] Procurement; [about 3 screens]. Available from: http://www.sadc.int/sadc-secretariat/directorates/office-deputy-executive-secretary-finance-administration/procurement/

[33] Southern African Development Community (SADC). Draft: SADC strategy for pooled procurement of essential medicines and health commodities: 2013–2017. Gaborne (Botswana): SADC; 2012 Available from: http://www.sarpam.net/wp-content/uploads/2012/12/SADC-PP-Strategy-16-11-12-final-English.pdf

[34] Republic of Mozambique; World Bank. Update of the country procurement assessment review (CPAR). Washington (DC): World Bank; 2008 Available from: http://documents.worldbank.org/curated/en/2008/06/16350125/mozambique-update-country-procurement-assessment-review-cpar

[35] Ghana Ministry of Health (MOH); DFID; World Health Organization. Ghana: assessment of medicines procurement and supply management systems in the public health sector: a country report 2009. Accra: MOH; 2009 Available from: http://apps.who.int/medicinedocs/documents/s18017en/s18017en.pdf

